# Prevalence, associated factors and consequence of problematic smartphone use among adolescents and young adults in Bangladesh: A cross-sectional study

**DOI:** 10.1371/journal.pone.0308621

**Published:** 2024-08-26

**Authors:** Md. Rabiul Islam, Archok Arigan Mondol, Ripa Kundu, Juliana Aditi Baroi, Sayma Akter, Taslima Jamal Urmi, A. S. M. Roknuzzaman, Md. Alamgir Hossain, Mohammad Masud Parves, Humair Bin Md. Omer, Eva Rahman Kabir

**Affiliations:** 1 School of Pharmacy, BRAC University, Merul Badda, Dhaka, Bangladesh; 2 Department of Pharmacy, University of Asia Pacific, Farmgate, Dhaka, Bangladesh; 3 Department of Pharmaceutical Chemistry, University of Dhaka, Dhaka, Bangladesh; 4 Department of Clinical Pharmacy and Pharmacology, University of Dhaka, Dhaka, Bangladesh; Virginia Tech Carilion School of Medicine, UNITED STATES OF AMERICA

## Abstract

**Background:**

Problematic smartphone use (PSU) and attention deficit hyperactivity disorder (ADHD) in children, adolescents, and young adults are of major concern to parents. However, the prevalence and associated factors related to these issues in Bangladeshi adolescents and young adults remain unclear to the best of our knowledge. The aim of this study is to assess PSU and ADHD in the context of adolescent and young adult age groups in Bangladesh.

**Methods:**

The present study collected data from diverse geographical locations in Bangladesh via face-to-face surveys using stratified random sampling methods. We considered age, sex, and geographic location stratification criteria. A total of 578 respondents participated in the survey initially. From this, we discarded 36 responses after screening because the information provided was insufficient or incomplete response. In the end, 542 replies were incorporated into the final analysis. PSU and ADHD depend on several factors, including the individual’s demographic background.

**Results:**

The prevalence of PSU and ADHD symptoms in adolescents and young adults in Bangladesh is 61.44% and 37.45%, respectively based on our findings. The symptoms of PSU are correlated with age, education level, family type (nuclear/joint), sleeping pattern, physical exercise, and residence area. ADHD symptoms are correlated with age, education level, living with family, smoking habit, physical disability, sleeping pattern, physical exercise, residence area, and PSU. Also, we observed that ADHD and PSU symptoms are positively correlated with each other.

**Conclusion:**

A large proportion of young adults and adolescents reported PSU and ADHD symptoms. The present findings have practical implications in clinical psychology, psychotherapy, and related policy considerations. We propose to develop an inclusive interventional strategy and community-based programs to address PSU and ADHD-related issues.

## 1. Introduction

A smartphone is a device that combines mobile phone and internet functionality [1]. Smartphones were first introduced to the general public in the year 2007 [2]. The inability of an individual (behavioral addiction) to regulate themselves in their personal smartphone use is known as problematic smartphone use (PSU). Certain features of PSU make it distinct from normal smartphone use e.g. individuals suffering from PSU exhibit withdrawal symptoms, tolerance development, dangerous use and behavioral problems. PSU has various labels such as smartphone dependency, PSU, and smartphone overuse. Individuals who struggle with PSU face physiological, psychological, and social problems. Adolescents and young adults are particularly vulnerable to PSU [1, 3]. Moreover, during the recent pandemic PSU has increased [4]. The use of social networking services is a significant factor in predicting PSU [1]. Excessive use of social media is often associated with psychological distress and thoughts of self-harm [5–7]. PSU is positively correlated with increased daily smartphone usage, social media usage, time spent playing games, poor sleep quality, and the intensity of depressive symptoms [8, 9]. Compared to older people, adolescents and young adults are more vulnerable to PSU. Their social media usage and smartphone use can expose them to negative peer feedback, distorted body image perceptions, online harassment, and constant unhealthy comparison with others [10].

The strongest warning signs of PSU are observed in those who got their first phone before turning thirteen [4]. Children who started using a cell phone at a young age exhibited increased behavioral issues, including nervousness, irritability, cognitive distraction, and laziness. These problems were further exacerbated over time [1, 11]. Studies on cell phone addiction revealed that smartphone use poses a greater risk of road accidents compared to alcohol abuse [4]. Another study suggests connections between PSU, physical health, and mental health issues. Musculoskeletal discomfort and sleep difficulties are the most prevalent issues associated with PSU [4]. Excessive smartphone use is often linked to hand dysfunction, cervical, neck, back, and shoulder problems, as well as poor postures like forward head posture [2]. Among students, PSU is associated with poor physical and mental fitness and academic performance [12]. Depression and sleep problems are prevalent psychological issues related to PSU among university students [8]. Previous research indicates that an individual’s psychological characteristics can be discerned by analyzing their patterns of smartphone usage [13].

The degree to which college students effectively manage their smartphone usage can significantly impact their mental health outcomes. According to research, college students are more likely to use smartphones adversely, which can have detrimental effects on their physical and mental health [14]. Remarkably, several psychological problems, such as anxiety, depression, bipolar disorder, dependent personality disorder, obsessive personality disorder, and somatization, are significantly correlated with PSU [4, 15]. Attention deficit hyperactivity disorder (ADHD) is a psychiatric condition characterized by persistent challenges in sustaining attention, hyperactivity, and impulsivity, and is estimated to impact 7% of young individuals.^19^ ADHD can also be exacerbated by PSU because of factors like irregular sleep schedules, extended screen time, fast graphic changes, cravings for instant gratification, and an insatiable thirst for pleasure [9, 16–18]. Research findings suggest that adolescents with ADHD are more likely to experience PSU, with a prevalence rate of 42.0%. Furthermore, adolescents with more severe ADHD symptoms tend to encounter social difficulties and exhibit lower levels of optimism [19, 20].

According to epidemiological studies, the incidence of ADHD in children and adolescents worldwide ranges from 5 to 10%, but the prevalence in adults is roughly 2.8%. Adolescents with ADHD face increased risks of school rejection, lower educational attainment, and challenges in peer relationships. Additionally, they show a higher tendency to engage in dangerous activities, including drug abuse, reckless driving, and deviant sexual behavior [21]. Adolescents with ADHD may be more attracted to smartphone use due to its instant response, rewards, and portability [16]. In univariable analyses, ADHD prevalence was higher in the Middle East than in North America. Multivariable analysis studies in Europe reported lower ADHD prevalence compared to North America [22]. Research suggests that individuals with PSU may struggle to focus or sustain attention, exhibiting symptoms similar to ADHD. Post-hoc analyses indicate that ADHD symptoms are higher in the high-risk PSU group compared to normal users. According to a different study, smartphones with access to the internet can make it more likely that people will develop an online addiction and weaken their capacity for sustained concentration [23]. A study conducted with adolescent smartphone addicts seeking psychiatric outpatient care in Korea revealed a high prevalence of PSU symptoms related to depression and ADHD. Approximately 40% of ADHD patients in the study were identified as smartphone addicts, and their vulnerability to addiction was attributed to poor attention and inhibition [24].

The tremendous development in popularity of smartphones has made them a central device for accessing information and services via the internet. The incidence of PSU in teenagers in Taiwan fluctuates from 13.9% to 25.7% [25]. A previous study conducted in Korea found that 4.9% of middle school students and 10.6% of university students had PSU [16]. In 2014, there were 39 million smartphone users in South Korea. Present estimates indicate that the number is 53.5 million, which is 97% of the total population of Korea [26]. Smartphones are easily accessible, leading to PSU in children and adolescents. According to a study, the prevalence rate of PSU was considerably greater in the ADHD group than it was in the non-ADHD group (34.4% vs. 15.4%). Additionally, the ADHD group showed more disruptive behavior and decreased everyday performance [27, 28]. Research from America, Europe, and the Middle East showed that the average adult rate of ADHD was 3.4%; high-income countries had a higher prevalence (4.2%) than low-income countries (1.9%). An epidemiological study conducted on 4,512 South Korean adolescents assessed the associations among PSU, ADHD symptoms, depression, and anxiety. The study found that students with PSU had a higher prevalence of ADHD symptoms (47.8%) compared to low-use smartphone users (19.7%) [29]. PSU hasn’t been officially recognized as a disorder worldwide, even though it has a significant impact on human physiology, social behavior, and psychology. A study in 2022 found that the estimated global prevalence rate of PSU is around 27%. In terms of research volume, China had the highest number of articles published on PSU in international academic journals. In Bangladesh, the number of social media users was 24.49 million in 2022, and it is projected to reach 33.6 million by 2027. On the other hand, not much research is conducted about how these platforms are used and how they affect mental health problems. Unfortunately, mental health issues in Bangladesh continue to be stigmatized and receive little attention [30–32]. The number of smartphone users has been rising worldwide, with the fastest increase occurring in Asia and Europe [16]. To the best of our knowledge studies on PSU and ADHD in South Asian countries, particularly in Bangladesh, are not being conducted adequately, despite the increasing prevalence of PSU [17, 18]. Furthermore, a clear relationship between PSU and ADHD remains unclear due to a lack of adequate data. PSU is a significant concern as it is closely associated with mental health issues.

The rationale of this study is to investigate the prevalence of PSU and symptoms of ADHD among young adults and adolescents in Bangladesh, as well as the potential factors that contribute to these conditions. Specifically, we investigated the importance of age, education level, living with family, sleeping timing, smoking habit, physical disability, physical exercise, and residence area of respondents in correlating with PSU and ADHD. We hope this study’s findings will help create a better understanding of PSU, ADHD symptoms, and associated factors that might aid create a comprehensive intervention plan to manage these mental health disorders.

## 2. Methods

### 2.1 Study design and participants

**Fig 1:**
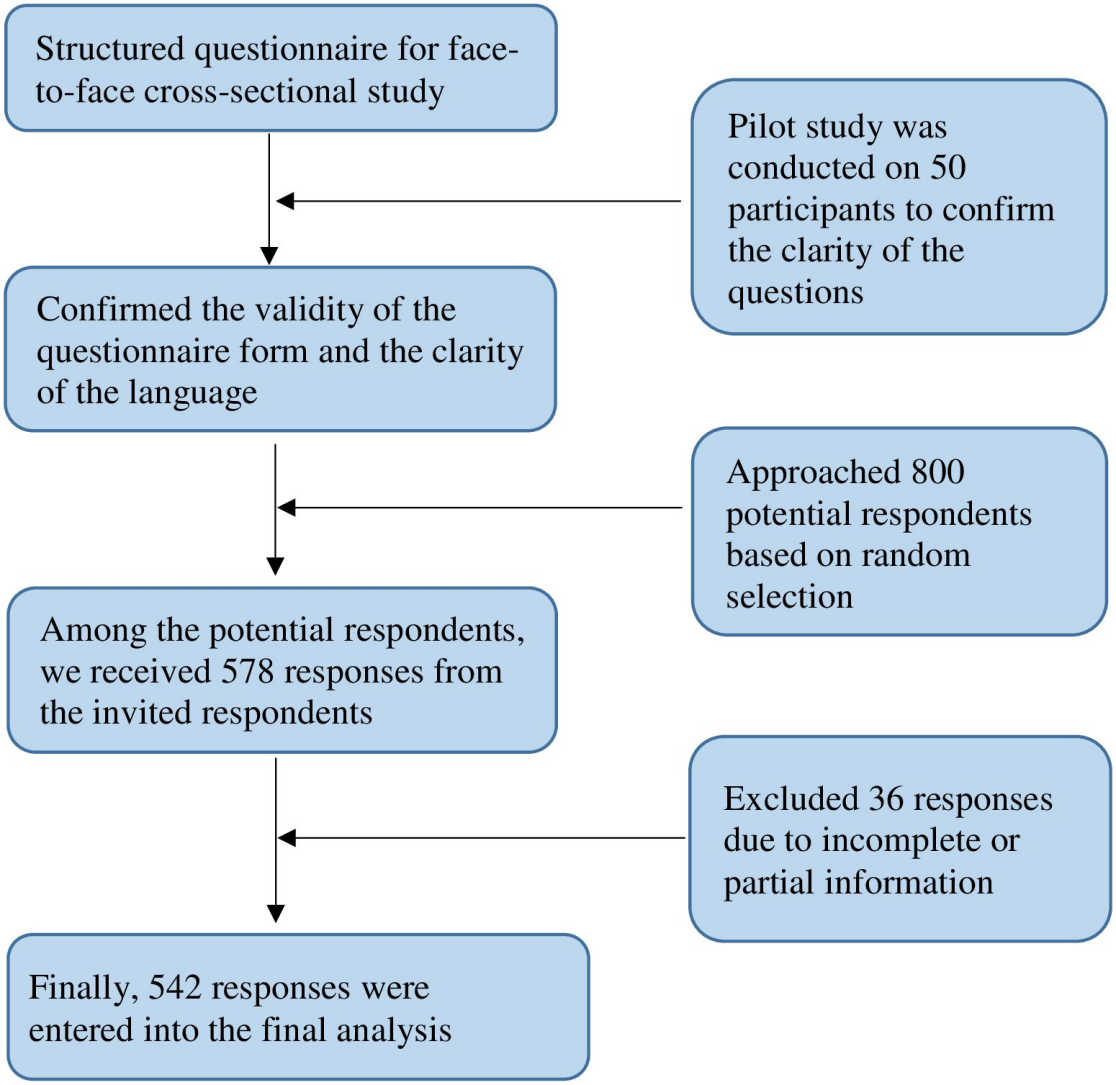
Flowchart showing of data collection.

Between January 1, 2023, and June 30, 2023, we performed this cross-sectional study among adolescents and young adults in Bangladesh. We assumed the response rate, confidence interval, and margin of error as 75%, 95%, and 5%, respectively. Based on the above assumptions, the present study required 384 responses. We applied stratified random sampling method to target potential respondents. We considered age, sex, and geographic location as stratification criteria. Initially, we approached 800 participants via face-to-face surveys. We got 578 responses from them, but after screening, 36 were excluded due to incomplete information. Finally, 542 responses (259 males and 283 females) were included in the final analysis (Fig 1). All participants live in Bangladesh and are of Bangladeshi descent. Before participation, they received an overview of the permission forms, eligibility requirements, procedures, and questionnaire. Once they were aware of the requirements for qualifying, they voluntarily provided their information. The volunteers received no payment for their participation in the study.

### 2.2 Estimations

We collected relevant socio-demographic information from the respondents using a pre-structured questionnaire. PSU and ADHD were assessed among the participants using globally recognized and validated scales. The questionnaires were first written in Bengali and then translated into English. The questions were translated with assistance by two proficient Bengali speakers, one with a medical background and the other with a non-medical background. An independent author integrated the translated versions to produce a Bengali-forward version, addressing inconsistencies. This Bengali version was then translated into two English questionnaires by professional medical translators. Another independent researcher combined the back-translated versions to produce a single English version. To make sure the questions were understandable and clear, a small, randomly selected sample was used for a pilot study. To ensure proper understanding, we offered the survey questionnaire in both Bengali and English.

### 2.3 Assessment of problematic smartphone use

The level of PSU was assessed using the short version of the smartphone addiction scale (SAS-SV), which is a globally recognized and validated scale with 10 questions. Each question is rated on a Likert scale from 1 to 6, representing “strongly disagree (1)” to “strongly agree (6).”. Participants rated how much they agree or disagree to ten questions on their current smartphone usage. The questions on this scale covered a range of issues related to addiction, including relationships focused on cyberspace, withdrawal symptoms, excessive usage, and tolerance, as well as disruptions in daily life [33]. One benefit of the SAS-SV is its ability to pinpoint a population that may be at a higher risk of developing PSU than others. The total score of SAS-SV can vary from 10 to 60. Higher scores on this scale indicate a higher level of PSU. The cut-off value for the SAS-SV score for this study was 32, with a sensitivity of 0.858 and a specificity of 0.874.

### 2.4 Assessment of attention deficit hyperactivity disorder

We used the ADHD self-report scale (ASRS) to assess ADHD among the participants. The ASRS consists of 18 items that align with the 18 DSM-4 criteria for ADHD. This scale was originally developed by the working group on ADHD at the World Health Organization (WHO). Participants rate themselves on a five-point Likert scale ranging from 0 to 4 (never, rarely, sometimes, often, very often). The first six items of ASRS are called screeners, indicating the frequency of their experiences of ADHD symptoms in the previous 6 months. An individual was deemed to be at an elevated risk for ADHD if they agreed with at least four of the six items. The remaining 12 additional questions are based on DSM-4 criteria to assess ADHD symptoms. Either the sum score of all questions can be computed or the first six questions (shortened version) are frequently used to assess the ADHD symptoms [34]. We calculated the total score for ASRS utilizing the scoring method recommended in the ASRS symptom checklist and screener manual. The total score of SAS-SV can vary from 0 to 72. The cut-off value for the ASRS score for this study was 39, with a sensitivity of 0.823 and a specificity of 0.857.

### 2.5 Statistical analyses

Microsoft Excel 2019 and IBM SPSS V.25.0, the Statistical Packages for Social Sciences, were used in the data analysis. Microsoft Excel was used to process the data. Excel file was then imported into IBM SPSS for additional analysis. We used descriptive statistics to find and compare the characteristics of the study participants. We used the chi-square test to compare between the groups. Additionally, logistic regression analysis was run to determine the primary factors associated with PSU. P-values less than 0.05 are considered the threshold for statistical significance.

### 2.6 Ethical consideration

The study was approved by the Ethical Review Committee of the University of Asia Pacific, Dhaka, Bangladesh (Ref: UAP/REC/2022/107). The ethical objective of the study was to ensure that the participants maintained complete clarity and transparency. We obtained written informed consent from the participants. In the case of minors (below 18 years), we obtained written parental consent. Additionally, the respondents were assured of their privacy. We also offered all participants the right to leave the study at any moment without any penalties. We conducted the present research in accordance with the guidelines specified in the Declaration of Helsinki.

## 3. Results

### 3.1 Description of the study participants

A total of 542 respondents below the age of 30 participated in the research. Among them, 52.21% were females, and 93.54% were unmarried and 61.81% were between the ages of 19 and 25 years. Among the participants, 61.44% had PSU symptoms, and 45.76% of the participants were from a medium-income family and non-smokers (90.59%). The majority of the participants were in secondary level of education (51.85%) and studies in Bengali medium (84.87%). A normal BMI was possessed by 63.66% of the respondents, whereas 24.35% were obese. About 81.37% of the study participants lived with their families, and around 74.35% of respondents were from urban areas. The majority of participants went to sleep between 12:01 AM and 2 AM, with a significant proportion getting 7–8 hours of sleep. Additionally, a large percentage of participants reported not engaging in regular physical exercise as well.

### 3.2 Prevalence and associated factors of problematic smartphone use

**Fig 2:**
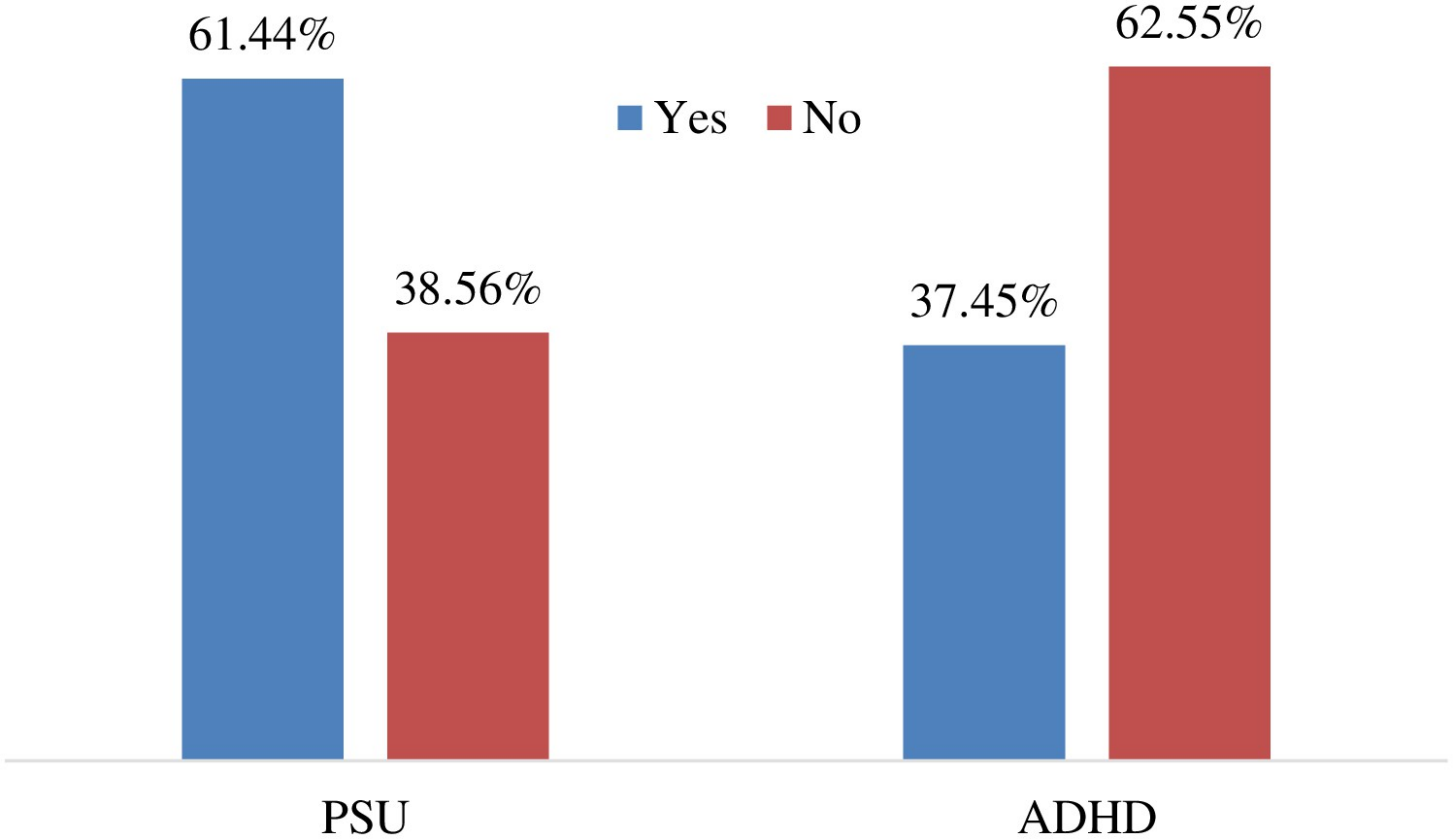
Prevalence of problematic smartphone use and attention-deficit hyperactivity disorder among the young adults and adolescents in Bangladesh”.

The prevalence of PSU symptoms among the participants was 61.44% (Fig 2). Our study finds that PSU significantly varies by age, education level, family type (nuclear/joint), sleeping timing, physical exercise, and residence area of respondents (Table 1). The proportions of respondents experiencing PSU symptoms were higher in (1) 26–30 years age group (76.92%) followed by 19–25 (64.78%) and 12–18 (53.03%). The proportion of participants who reported PSU symptoms from nuclear and joint families was 64.58% and 53.80%, respectively. Proportions of respondents with or without PSU symptoms having primary, secondary, graduation, master’s, or above the level of education were 45.45% vs. 54.55%, 46.62% vs. 53.38%, 30.13% vs. 69.87%, and 19.05% vs. 80.95%, respectively. We noticed the proportion of participants who reported PSU symptoms with or without being actively involved in physical exercise were 48.43% vs. 66.84%, respectively. Proportions of respondents with or without PSU symptoms between urban and rural areas were 66.50% vs. 46.76%, respectively. Also, we observed the prevalence of PSU was higher among individuals who go to sleep after midnight, need more time to fall asleep and wake up late. The Cronbach’s alpha for SAS-SV among the responses was 0.814 that confirms good reliability and internal consistency of PSU measures.

**Table 1:** Proportion of participants reported symptoms of smartphone addiction and its association with demographic variables.

	Symptoms of smartphone addiction		χ^2^	df	p-value
	No	Yes			
Age (in years)					
12–18	85 (46.96)	96 (53.04)	9.597	2	**0.008**
19–25	118 (35.22)	217 (64.78)			
26–30	6 (23.08)	20 (76.92)			
Sex					
Male	104 (40.15)	155 (59.85)	0.532	1	0.466
Female	105 (37.10)	178 (62.90)			
BMI (kg/m^2^)					
CED (<18.5)	30 (46.15)	35 (53.85)	7.679	2	0.021
Normal (18.5–25.0)	141 (40.87)	204 (59.13)			
Obese (>25.0)	38 (28.79)	94 (71.21)			
Level of education					
Primary	5 (45.45)	6 (54.55)	18.167	3	**<0.001**
Secondary	131 (46.62)	150 (53.38)			
Graduation	69 (30.13)	160 (69.87)			
Master’s or above	4 (19.05)	17 (80.95)			
Medium of education					
Bangla	184 (40.00)	276 (60.00)	2.658	1	0.103
English	25 (30.49)	57 (69.51)			
Marital status					
Married	15 (42.86)	20 (57.14)	0.292	1	0.589
Unmarried	194 (38.26)	313 (61.74)			
Family type					
Nuclear	136 (35.42)	248 (64.58)	5.497	1	**0.019**
Joint	73 (46.20)	85 (53.80)			
Family structure					
Both parents	189 (38.03)	308 (61.97)	2.136	2	0.344
Single parent	19 (47.50)	21 (52.50)			
Adopted	1 (20.00)	4 (80.00)			
Currently living with family					
Yes	179 (40.59)	262 (59.41)	4.111	1	0.043
No	30 (29.70)	71 (70.30)			
Time of going bed at night					
Before 10:00 PM	35 (54.69)	29 (45.31)	12.148	3	**0.007**
10:00 PM to 12:00 AM	67 (41.87)	93 (58.13)			
12:01 AM to 2 AM	75 (31.91)	160 (68.09)			
After 2:00 AM	32 (38.55)	51 (61.45)			
Time needs to fall asleep					
Less than 15 minutes	100 (48.08)	108 (51.92)	13.541	3	**0.004**
15–30 minutes	62 (31.00)	138 (69.00)			
31–60 minutes	28 (34.15)	54 (65.85)			
More than 60 minutes	19 (36.54)	33 (63.46)			
Time of waking up					
Before 5:00 AM	23 (48.94)	24 (51.06)	14.488	3	**0.002**
5:00AM to 7:00 AM	81 (48.21)	87 (51.79)			
7:00 AM to 9:00 AM	67 (32.06)	142 (67.94)			
After 9:00 AM	38 (32.20)	80 (67.80)			
Duration of sleep at night					
Less than 4 hours	16 (45.71)	19 (54.29)	7.514	3	0.057
4–6 hours	73 (34.11)	141 (65.89)			
7–8 hours	102 (43.97)	130 (56.03)			
More than 8 hours	18 (29.51)	43 (70.49)			
Time spent in bed					
Less than 5 hours	50 (45.45)	60 (54.55)	7.585	3	0.055
5–7 hours	84 (42.21)	115 (57.79)			
8–10 hours	56 (31.28)	123 (68.72)			
More than 10 hours	19 (35.19)	35 (64.81)			
Economic impression					
Low	90 (42.65)	121 (57.35)	2.760	2	0.252
Medium	87 (35.08)	161 (64.92)			
High	32 (38.55)	51 (61.45)			
Smoking habit					
Nonsmoker	191 (38.90)	300 (61.10)	0.254	1	0.615
Smoker	18 (35.29)	33 (64.71)			
Visual performance					
Aided	67 (36.22)	118 (63.78)	0.652	1	0.419
Non-aided	142 (39.78)	215 (60.22)			
Presence of physical disability					
Yes	12 (32.43)	25 (67.57)	0.630	1	0.428
No	197 (39.01)	308 (60.99)			
Regular physical exercise					
Yes	82 (51.57)	77 (48.43)	16.079	1	**<0.001**
No	127 (33.16)	256 (66.84)			
Residence area					
Rural	74 (53.24)	65 (46.76)	16.997	1	**<0.001**
Urban	135 (33.50)	268 (66.50)			

Abbreviation: df, degree of freedom; BMI, body mass index; CED, chronic energy deficiency; χ^2^, chi-square.

### 3.3 Prevalence and associated factors of attention deficit hyperactivity disorder

We observed that the prevalence of ADHD symptoms among the participants was 37.45% (Fig 2). The prevalence of ADHD symptoms significantly varied by age, education level, living with family (yes/no), sleeping timing, smoking habit, physical disability, physical exercise, residence area, and PSU symptoms of respondents (Table 2). There was a greater percentage of responders reporting symptoms of ADHD in (1) the 26–30-year-old age group (76.92%), followed by 19–25 (40.30%), and 12–18 (26.52%). The proportion of participants living with or without families and showing ADHD symptoms was 35.37% and 46.53%, respectively. Proportions of respondents with or without ADHD symptoms having primary, secondary, graduation, master’s or above level of education were 45.45% vs. 54.55%, 30.25% vs. 69.75%, 44.10% vs. 55.90%, and 57.14% vs. 42.86%, respectively. We noticed the proportion of participants who reported ADHD symptoms was higher among individuals with smoking habits (54.90%) and physical disabilities (54.04%). Also, we observed the prevalence of ADHD symptoms was higher among individuals who go to sleep after midnight, need more time to fall asleep, wake up late in the morning, and spend more time in bed. The Cronbach’s alpha for ASRS among the responses was 0.798 that also confirms good reliability and internal consistency of ADHD measures.

**Table 2:** Proportion of participants reported symptoms of attention deficit hyperactivity disorder and its association with demographic variables.

	Symptoms of attention deficit hyperactivity disorder		χ^2^	df	p-value
	No	Yes			
Age (in years)					
12–18	133 (73.48)	48 (26.52)	27.685	2	**<0.001**
19–25	200 (59.70)	135 (40.30)			
26–30	6 (23.08)	20 (76.92)			
Sex					
Male	177 (68.34)	82 (31.66)	7.108	1	0.008
Female	162 (57.24)	121 (42.76)			
BMI (kg/m^2)^					
CED (<18.5)	41 (63.08)	24 (36.92)	1.344	2	0.511
Normal (18.5–25.0)	221 (64.06)	124 (35.94)			
Obese (>25.0)	77 (58.33)	55 (41.67)			
Level of education					
Primary	6 (54.55)	5 (45.45)	14.326	3	**0.002**
Secondary	196 (69.75)	85 (30.25)			
Graduation	128 (55.90)	101 (44.10)			
Master’s or above	9 (42.86)	12 (57.14)			
Medium of education					
Bangla	290 (63.04)	170 (36.96)	0.321	1	0.571
English	49 (59.76)	33 (40.24)			
Marital status					
Married	18 (51.43)	17 (48.57)	1.974	1	0.160
Unmarried	321 (63.31)	186 (36.69)			
Family type					
Nuclear	240 (62.50)	144 (37.50)	0.001	1	0.972
Joint	99 (62.66)	59 (37.34)			
Family structure					
Both parents	313 (62.98)	184 (37.02)	0.488	2	0.783
Single parent	23 (57.50)	17 (42.50)			
Adopted	3 (60.00)	2 (40.00)			
Living with family					
Yes	285 (64.63)	156 (35.37)	4.369	1	**0.037**
No	54 (53.47)	47 (46.53)			
Time of going bed at night					
Before 10:00 PM	53 (82.81)	11 (17.19)	17.42	3	**0.001**
10:00 PM to 12:00 AM	105 (65.63)	55 (34.37)			
12:01 AM to 2 AM	138 (58.72)	97 (41.28)			
After 2:00 AM	43 (51.81)	40 (48.19)			
Time needs to fall asleep					
Less than 15 minutes	146 (70.19)	62 (29.81)	9.529	3	**0.023**
15–30 minutes	120 (60.00)	80 (40.00)			
31–60 minutes	44 (53.66)	38 (46.34)			
More than 60 minutes	29 (55.77)	23 (44.23)			
Time of waking up					
Before 5:00 AM	33 (70.21)	14 (29.79)	12.773	3	**0.005**
5:00AM to 7:00 AM	121 (72.02)	47 (27.98)			
7:00 AM to 9:00 AM	120 (57.42)	89 (42.58)			
After 9:00 AM	65 (55.08)	53 (44.92)			
Duration of sleep at night					
Less than 4 hours	20 (57.14)	15 (42.86)	0.785	3	0.853
4–6 hours	132 (61.68)	82 (38.32)			
7–8 hours	149 (64.22)	83 (35.78)			
More than 8 hours	38 (62.30)	23 (37.70)			
Time spent in bed					
Less than 5 hours	72 (65.45)	38 (34.55)	9.184	3	**0.027**
5–7 hours	136 (68.34)	63 (31.66)			
8–10 hours	105 (58.66)	74 (41.34)			
More than 10 hours	26 (48.15)	28 (51.85)			
Economic impression					
Low	138 (65.40)	73 (34.60)	1.379	2	0.502
Medium	149 (60.08)	99 (39.92)			
High	52 (62.65)	31 (37.35)			
Smoking habit					
Nonsmoker	316 (64.36)	175 (35.64)	7.316	1	**0.007**
Smoker	23 (45.10)	28 (54.90)			
Visual performance					
Aided	105 (56.76)	80 (43.24)	4.019	1	0.054
Non-aided	234 (65.55)	123 (34.45)			
Presence of physical disability					
Yes	17 (45.95)	20 (54.05)	4.671	1	**0.031**
No	322 (63.76)	183 (36.24)			
Regular physical exercise					
Yes	114 (71.70)	45 (28.30)	8.045	1	**0.005**
No	225 (58.75)	158 (41.25)			
Residence area					
Rural	108 (77.70)	31 (22.30)	18.32	1	**<0.001**
Urban	231 (57.32)	172 (42.68)			
Problematic smartphone use					
No	165 (78.95)	44 (21.05)	39.062	1	**<0.001**
Yes	174 (52.25)	159 (47.75)			

Abbreviation: df, degree of freedom; BMI, body mass index; CED, chronic energy deficiency; χ^2^, chi-square.

### 3.4 Regression analysis

Binary logistic regression analyses were used to ascertain the relationship between PSU and ADHD symptoms with sociodemographic profile of participants (Table 3). Those who are in the secondary level of education reported being 0.213 times less likely than those with a master’s degree or above to experience PSU symptoms (OR = 0.213; 95% CI = 0.048–0.954, p = 0.043). Married respondents were 0.387 times less likely to get addicted to smartphones compared to unmarried respondents (OR = 0.387; 95% CI = 0.153–0.974, p = 0.044). Respondents engaged in physical activity were 0.663 times less likely to suffer from PSU compared to physically inactive individuals (OR = 0.663, 95% C = 0.412–1.067, p = 0.090). Respondents residing in rural areas are 0.364 times less likely to develop PSU compared to urban residents (OR = 0.364, 95% C = 0.170–0.779, p = 0.009). The chances of developing ADHD symptoms were found to be 0.190 times lower in those aged 19–25 than in individuals aged between 26–30 (OR = 0.190; 95% CI = 0.050–0.725, p = 0.015). Males had a 0.510 times lower prevalence of ADHD symptoms compared to females (OR = 0.510; 95% CI = 0.319–0.816, p = 0.005). Our data on gender differences in ADHD symptoms is in contrast to previous literature studies where males exhibited higher prevalence of ADHD symptoms than females. However, such a conclusion is not definite and still under active research studies as exemplified by the work of Rucklidge et. al. who found that gender differences in ADHD symptoms are somewhat more similar than different [35]. Moreover, such gender differences might be demographic dependent altogether as might be in the case of the Bangladeshi youth population. Non-smokers are 0.321 times less likely to develop ADHD symptoms compared to smokers (OR = 0.190; 95% CI = 0.155–0.667, p = 0.002). Also, we noticed that PSU and ADHD symptoms were positively correlated with each other (r = 468, p<0.001).

**Table 3:** Logistic regression analysis of demographic variables for symptoms of problematic smartphone use and attention deficit hyperactivity disorder.

Characteristics	OR	PSU (95% CI)		p-value	OR	ADHD (95% CI)		p-value
		Lower bound	Upper bound			Lower bound	Upper bound	
Age (in years)								
12–18	1.488	0.339	6.531	0.598	0.220	0.047	1.028	0.054
19–25	0.625	0.175	2.231	0.469	0.190	0.050	0.725	**0.015**
26–30	1				1			
Sex								
Male	1.154	0.740	1.798	0.528	0.510	0.319	0.816	**0.005**
Female	1				1			
BMI (kg/m^2^)								
CED (<18.5)	0.581	0.271	1.245	0.163	1.554	0.702	3.440	0.276
Normal (18.5–25.0)	0.770	0.462	1.282	0.315	1.109	0.667	1.844	0.691
Obese (>25.0)	1				1			
Level of education								
Primary	0.177	0.022	1.391	0.100	3.177	0.360	28.060	0.298
Secondary	0.213	0.048	0.954	**0.043**	2.614	0.542	12.599	0.231
Graduation	0.406	0.097	1.701	0.218	2.746	0.621	12.149	0.183
Master’s or above	1				1			
Medium of education								
Bangla	1.044	0.559	1.949	0.893	1.362	0.738	2.513	0.323
English	1				1			
Marital status								
Married	0.387	0.153	0.974	**0.044**	2.247	0.876	5.766	0.092
Unmarried	1				1			
Family type								
Nuclear	1.384	0.883	2.170	0.156	0.729	0.450	1.181	0.199
Joint	1				1			
Family structure								
Both parents	0.149	0.012	1.915	0.144	1.531	0.116	20.241	0.746
Single parent	0.132	0.009	1.887	0.136	2.391	0.162	35.201	0.525
Adopted	1				1			
Currently living with family								
Yes	0.807	0.448	1.453	0.474	0.966	0.546	1.710	0.906
No	1				1			
Time of going bed at night								
Before 10:00 PM	1.614	0.584	4.461	0.356	0.386	0.120	1.241	0.110
10:00 PM to 12:00 AM	1.170	0.552	2.481	0.682	0.748	0.347	1.615	0.460
12:01 AM to 2 AM	1.658	0.861	3.192	0.130	0.690	0.357	1.334	0.270
After 2:00 AM	1				1			
Time needs to fall asleep								
Less than 15 minutes	0.767	0.345	1.704	0.515	0.892	0.392	2.027	0.785
15–30 minutes	1.361	0.610	3.040	0.452	0.822	0.366	1.846	0.634
31–60 minutes	0.906	0.372	2.210	0.829	1.099	0.449	2.693	0.836
More than 60 minutes	1				1			
Time of waking up								
Before 5:00 AM	0.749	0.290	1.934	0.550	1.387	0.488	3.943	0.539
5:00AM to 7:00 AM	0.625	0.318	1.228	0.172	0.870	0.430	1.760	0.698
7:00 AM to 9:00 AM	1.031	0.560	1.897	0.923	1.154	0.628	2.118	0.645
After 9:00 AM	1				1			
Duration of sleep at night								
Less than 4 hours	0.447	0.154	1.295	0.138	1.065	0.348	3.259	0.913
4–6 hours	0.975	0.442	2.150	0.949	1.202	0.542	2.663	0.651
7–8 hours	0.509	0.251	1.034	0.062	1.266	0.605	2.649	0.532
More than 8 hours	1				1			
Time spent in bed								
Less than 5 hours	0.888	0.386	2.042	0.779	0.444	0.189	1.042	0.062
5–7 hours	0.924	0.425	2.011	0.842	0.387	0.175	0.854	0.019
8–10 hours	1.606	0.740	3.482	0.231	0.483	0.225	1.037	0.062
More than 10 hours	1				1			
Economic impression								
Low	1.072	0.570	2.018	0.829	1.144	0.590	2.217	0.691
Medium	0.994	0.542	1.824	0.984	0.923	0.493	1.729	0.802
High	1				1			
Smoking habit								
Nonsmoker	0.876	0.424	1.807	0.720	0.321	0.155	0.667	**0.002**
Smoker	1				1			
Visual performance								
Aided	0.697	0.422	1.152	0.160	1.079	0.658	1.772	0.762
Non-aided	1				1			
Presence of physical disability								
Yes	0.937	0.407	2.153	0.877	1.692	0.771	3.717	0.190
No	1				1			
Regular physical exercise								
Yes	0.663	0.412	1.067	**0.090**	0.862	0.508	1.464	0.583
No	1				1			
Residence area								
Rural	0.364	0.170	0.779	**0.009**	0.733	0.327	1.644	0.451
Urban	1				1			

Abbreviation: df, degree of freedom; BMI, body mass index; CED, chronic energy deficiency; CI, confidence interval; ADHD, attention deficit hyperactivity disorder; PSU, problematic smartphone use; OR, odds ratio.

## 4. Discussion

With over 165.5 million inhabitants, Bangladesh is one of the most densely populated countries in the world, which has contributed to the steady rise in internet usage [36]. In Bangladesh, social media usage has increased dramatically, but there haven’t been much in-depth studies on how these platforms affect mental health [37, 38]. Lately, a few studies have found that young individuals who use social networking sites for more than two hours a day frequently have poor mental health, psychological discomfort, suicidal thoughts, and unmet psychiatric requirements [39–41]. Mental illnesses have high impact on the public health system and significantly contribute to the worldwide disease burden [42–46]. It is regrettable that the stigma and attention associated towards mental health issues still persists in Bangladesh [47]. According to the WHO assessment, only 7.7% of people in Bangladesh with mental health issues receive the appropriate care leaving a large volume of mental health patients with treatment gap [48].

Our current work thoroughly analyzed PSU symptoms, associated factors and the connection between PSU and ADHD symptoms among adolescents and young adults. Results indicate that there is a strong correlation between symptoms ADHD and PSU in adolescents and young adults. The study also identified several factors that influence PSU and ADHD symptoms such as education level, gender role, physical activity, marital status, residence area, smoking habits, sleeping time and living status. Based on these factors, preliminary interventionist methods to overcome or reduce PSU symptoms in children and young adults might be advised but further studies are required to validate treatment options since the cause for PSU are not fully understood yet. For example, we suggest children and young adults may engage in more social interactions and actively involve friends and family members in their activities that might have some positive spillover effect to counter PSU. Limiting smartphone usage and dedicating more time to outdoor games and physical exercise might be beneficial as well. Effective time management might play a crucial role in combating PSU. Alongside internet usage, we suggest adults might create schedules that prioritize a variety of activities, which might be likely to reduce the urge to constantly check their phones. We also advise seeking counseling as an option for adolescents to express their emotions and address the underlying causes of excessive online behavior. Individuals with mental health conditions aren’t aware of the fact that PSU can significantly contribute to their mental health problems. Additionally, both urban and rural populations in Bangladesh are increasingly susceptible to mental health problems and social hazards [49–53]. We expect that our study will help raise awareness on PSU and the mental health status, specifically ADHD, of the Bangladeshi population.

According to the findings, female students exhibit a higher frequency of mobile phone usage compared to males. The results of this study align with previous research, indicating a significant correlation between female gender and mobile phone addiction. An earlier study emphasized that gender is a crucial factor in predicting cell phone addiction, highlighting that women are more prone to becoming addicted than men [54]. Moreover, several studies have shown that women have higher rates of mobile phone dependence and problematic use compared to men [55–57]. This may be explained by the fact that women are more likely to have PSUs due to their demographic profiles and social structure. Another study highlighted the association between virtual spaces, such as mobile phones and the internet, with conditions like depression, anxiety, dependency, and addiction [15, 57]. Other research has demonstrated that residing in a nuclear family is also associated with PSU [38]. In line with prior studies, this investigation discovered that PSU is more likely to be linked with sedentary behavior and being overweight or obese [38, 58, 59]. Other studies have also indicated no correlation between smartphone use and sleep duration [39, 60, 61]. A Korean longitudinal study by Lee et al. discovered that PSU is associated with worse sleep quality but has no effect on how long people sleep [39, 60]. In this study, PSU emerged as one of the most significant predictors of ADHD, consistent with findings from other literature studies [39,58, 62]. There was a strong correlation found between symptoms of ADHD and poor sleep quality [63]. Addicts to smartphones have trouble focusing or maintaining their attention, and they often show symptoms of ADHD [23, 64].

The prevalence of PSU and ADHD are increasing worldwide, and the COVID-19 pandemic has augmented these issues. Many earlier studies report that adolescents and young adults in Bangladesh are suffering from several mental health issues. The present study revealed that a large proportion of adolescents and young adults in Bangladesh reported PSU and ADHD symptoms. A number of demographic and lifestyle-related factors were estimated as associated risk factors for PSU and ADHD among adolescents and young adults based on logistic regression model. The present findings highlight the importance of PSU and ADHD in creating a context-specific, inclusive interventional approach, adding to the body of evidence already in existing literature. Addressing PSU and ADHD could play a significant role in mitigating related mental health problem of adolescents and young adults in Bangladesh. This study has the potential to convey a message to the world regarding the smartphone usage and ADHD symptoms of the Bangladeshi population. The results of this study have wide-ranging implications for psychology, health, and academia. These findings can be used by parents and educational institutions to help youth, especially those with mental health issues. Regulatory organizations can consider implementation strategies, such as server access control during specific times (frequently at night), to mitigate addictive smartphone usage. Psychotherapists can also derive valuable insights from these studies to enhance their treatment approaches. The government may also adopt policies to prioritize the mental health of young adults to safeguard their well-being.

This study has several significant points. Firstly, it examines the connection between PSU and ADHD, taking into account demographic profiles among young people in Bangladesh. Secondly, the study aims to understand the association between PSU and ADHD. Thirdly, the data collection from across Bangladesh allowed for diverse socioeconomic backgrounds. The translated Bangla questionnaire ensured a proper understanding of the questions. Lastly, the findings of this study have broader implications for future context-specific research on PSU and ADHD, highlighting the importance of comprehensive mental health assessment and further investigation among young adults in Bangladesh.

Our present study has some limitations as well and we would like to discuss these limitations here. Firstly, the cross-sectional nature of the study is not applicable for the impact measurement of the PSU related parameters we found over time. As mentioned in the introduction, future work in PSU and ADHD symptoms using a longitudinal study approach is imperative and would provide further validation of our current work. Secondly, although the current study found signification correlation between PSU and factors e.g. education level, family status, gender roles, residence area, sleeping habits etc., the potential working mechanisms (routes) via which these variables affect PSU is not examined here and beyond the scope of our present investigation. Thirdly, we assessed PSU and ADHD symptoms only among the young generations in Bangladesh and a natural extrapolation would be to assess these underexplored diseases to other age groups in the country as well. Finally, the piloting of the study sometimes does not provide accurate information about the understanding of questions among the respondents.

## 5. Conclusion

In our current study, we have observed a substantial prevalence of PSU among young adults with ADHD in Bangladesh. Therefore, in Bangladesh, the promotion of mental health among children, adolescents, and young people must be given top priority by healthcare authorities and policymakers. Our results highlight the necessity for all-encompassing approaches to address the issues related to ADHD and PSU on a national and worldwide scale. To decrease excessive smartphone use, national and international public health organizations should work together to produce policies and guidelines. Furthermore, it is necessary to increase public awareness of the possible long-term effects PSU on young adults by community-based programs. We suggest education clinical psychiatrists and psychotherapists need to take this into account while interacting with patients and carrying out intervention procedures. However, further research with enhanced study designs is necessary to confirm these preliminary results.

## Supporting information

S1 Checklist (DOCX)

S1 File (DOCX)
