# Supplementary material for: Prevalence, associated factors and consequence of problematic smartphone use among adolescents and young adults in Bangladesh: A cross-sectional study
Source: PLoS One. 2024 Aug 26;19(8):e0308621. doi: 10.1371/journal.pone.0308621 (PMC11346645; doi:10.1371/journal.pone.0308621)
Supplement: S1 Checklist — (DOCX) [file pone.0308621.s001.docx]

**Title: Prevalence and factors associated with problematic smartphone use and attention deficit hyperactivity disorder among the adolescents and young adults in Bangladesh**

STROBE Statement—Checklist of items that should be included in reports of ***cross-sectional studies***

|  | | Item No | Recommendation | Section/Paragraph | |
| --- | --- | --- | --- | --- | --- |
| **Title and abstract** | | 1 | (*a*) Indicate the study’s design with a commonly used term in the title or the abstract | Title (page 1) | |
|  |  |  | (*b*) Provide in the abstract an informative and balanced summary of what was done and what was found | Abstract (page 2) | |
| Introduction | | | | | |
| Background/rationale | | 2 | Explain the scientific background and rationale for the investigation being reported | Introduction  Paragraphs 1-4 (Page 3-6) | |
| Objectives | | 3 | State specific objectives, including any prespecified hypotheses | Introduction  Paragraphs 5 (Page 6) | |
| Methods | | | | | |
| Study design | | 4 | Present key elements of study design early in the paper | Methods  Paragraphs 1 (Page 6) | |
| Setting | | 5 | Describe the setting, locations, and relevant dates, including periods of recruitment, exposure, follow-up, and data collection | Methods  Paragraphs 1 (Page 6) | |
| Participants | | 6 | (*a*) Give the eligibility criteria, and the sources and methods of case ascertainment and control selection. Give the rationale for the choice of cases and controls | Methods  Paragraphs 1 (Page 6) | |
|  |  |  | (*b*) For matched studies, give matching criteria and the number of controls per case | N/A | |
| Variables | | 7 | Clearly define all outcomes, exposures, predictors, potential confounders, and effect modifiers. Give diagnostic criteria, if applicable | Methods  Paragraphs 2 (Page 7) | |
| Data sources/ measurement | | 8* | For each variable of interest, give sources of data and details of methods of assessment (measurement). Describe comparability of assessment methods if there is more than one group | Methods  Paragraphs 2-3  (Page 6-7) | |
| Bias | | 9 | Describe any efforts to address potential sources of bias | Methods  Paragraphs 1 (Page 6) | |
| Study size | | 10 | Explain how the study size was arrived at | Methods  Paragraphs 1 (Page 6) | |
| Quantitative variables | | 11 | Explain how quantitative variables were handled in the analyses. If applicable, describe which groupings were chosen and why | Methods  Paragraphs 1 (Page 7) | |
| Statistical methods | | 12 | (*a*) Describe all statistical methods, including those used to control for confounding | Methods  Paragraphs 5  (Page 8) | |
|  |  |  | (*b*) Describe any methods used to examine subgroups and interactions | Methods  Paragraphs 5  (Page 8) | |
|  |  |  | (*c*) Explain how missing data were addressed | Methods  Paragraphs 1 (Page 6) | |
|  |  |  | (*d*) If applicable, explain how matching of cases and controls was addressed | N/A | |
|  |  |  | (*e*) Describe any sensitivity analyses | N/A | |
| Results | | | | | |
| Participants | | 13* | (a) Report numbers of individuals at each stage of study—eg numbers potentially eligible, examined for eligibility, confirmed eligible, included in the study, completing follow-up, and analysed | Results  Paragraphs 1  (Page 8-9) | |
|  |  |  | (b) Give reasons for non-participation at each stage | N/A | |
|  |  |  | (c) Consider use of a flow diagram | N/A | |
| Descriptive data | | 14* | (a) Give characteristics of study participants (eg demographic, clinical, social) and information on exposures and potential confounders | Results  Paragraphs 2  (Page 9) | |
|  |  |  | (b) Indicate number of participants with missing data for each variable of interest | N/A | |
| Outcome data | | 15* | Report numbers in each exposure category, or summary measures of exposure | Results  Paragraphs 2  (Page 9) | |
| Main results | | 16 | (*a*) Give unadjusted estimates and, if applicable, confounder-adjusted estimates and their precision (eg, 95% confidence interval). Make clear which confounders were adjusted for and why they were included | Results  Paragraphs 2-4  (Page 9-10)  Table 1, 2, 3 |  |
|  |  |  | (*b*) Report category boundaries when continuous variables were categorized | N/A |  |
|  |  |  | (*c*) If relevant, consider translating estimates of relative risk into absolute risk for a meaningful time period | N/A |  |
| Other analyses | 17 | Report other analyses done—eg analyses of subgroups and interactions, and sensitivity analyses | | N/A |  |
| Discussion | | | | |  |
| Key results | 18 | Summarise key results with reference to study objectives | | Discussion  Paragraph 1  (page 10) |  |
| Limitations | 19 | Discuss limitations of the study, taking into account sources of potential bias or imprecision. Discuss both direction and magnitude of any potential bias | | Strengths and limitations section  (page 12) |  |
| Interpretation | 20 | Give a cautious overall interpretation of results considering objectives, limitations, multiplicity of analyses, results from similar studies, and other relevant evidence | | Discussion  Paragraph 1-3  (page 10-12) |  |
| Generalisability | 21 | Discuss the generalisability (external validity) of the study results | | Discussion  Paragraph 2-3  (page 11-12) |  |
| Other information | | | | |  |
| Funding | 22 | Give the source of funding and the role of the funders for the present study and, if applicable, for the original study on which the present article is based | | Page 14 |  |

*Give information separately for cases and controls in case-control studies and, if applicable, for exposed and unexposed groups in cohort and cross-sectional studies.

**Note:** An Explanation and Elaboration article discusses each checklist item and gives methodological background and published examples of transparent reporting. The STROBE checklist is best used in conjunction with this article (freely available on the Web sites of PLoS Medicine at http://www.plosmedicine.org/, Annals of Internal Medicine at http://www.annals.org/, and Epidemiology at http://www.epidem.com/). Information on the STROBE Initiative is available at www.strobe-statement.org.
