# Supplementary material for: Prevalence, associated factors and consequence of problematic smartphone use among adolescents and young adults in Bangladesh: A cross-sectional study
Source: PLoS One. 2024 Aug 26;19(8):e0308621. doi: 10.1371/journal.pone.0308621 (PMC11346645; doi:10.1371/journal.pone.0308621)
Supplement: S1 File — (DOCX) [file pone.0308621.s002.docx]

Assessment of problematic smartphone use and attention deficit disorder among adolescents and young adults in Bangladesh

**Please tick the box for each statement**

*Express your consent to participate in the research and processing of anonymous data for scientific purposes.

- - Agree
  - Disagree

**Section 1: Questions about sociodemographic information**

1. *Age in years

- 12-18
- 19-25
- 26-30

2. *Sex

- Male
- Female

3. *Weight in kg

- …….

4. *Height, for example if the hight is 5 feet 2 inch, plese write 5.2.

- …….

1. *Level of education

- Primary
- Secondary
- Graduation
- Masters or above

1. *Medium of education

- Bangla
- English

1. *Marital status

- Unmarried
- Married

1. *Family type

- Joint
- Nuclear

1. *Family structure

- Both parents
- Single parent
- Adopted

1. *Living with family

- Yes
- No

1. * When do you usually go to bed for sleep at night?
   - Before 10.00 PM
   - 10.01 PM to 12.00 AM
   - 12.01 AM to 2.00 AM
   - After 2.00 AM
2. * How long (in minutes) has it take you to fall asleep each night?

- Less than 15 minutes
- 15-30 minutes
- 31-60 minutes
- More than 60 minutes

1. * When have you usually gotten up in the morning?

- Before 5.00 AM
- 5.00 AM to 7.00 AM
- 7.01 AM to 9.00 AM
- After 9.00 AM

1. * How many hours of actual sleep did you get at night?

- Less than 4 hours
- 4 to 6 hours
- 7 to 8 hours
- More than 8 hours

1. * How many hours do you spend in bed?

- Less than 5 hours
- 5 to 7 hours
- 8 to 10 hours
- More than 10 hours

1. *Economic impression

- Low
- Medium
- High

1. *Smoking habit

- Smoker
- Nonsmoker

1. *Do you use any glass with power to see and read?

- Yes
- No

1. Do you have any other physical disability or disease?

- Yes
- No

1. *Do you perform physical exercise regularly?

- Yes
- No

1. *Area of residence

- Urban
- Rural

***Section 2 : Smartphone addiction scale-short version (SAS-SV)***

1. Missing planned work due to smartphone use

- Strongly disagree
- Disagree
- Weakly disagree
- Weakly agree
- Agree
- Strongly agree

2. Having a hard time concentrating in class, while doing assignments, or while working due to smartphone use.

- Strongly disagree
- Disagree
- Weakly disagree
- Weakly agree
- Agree
- Strongly agree

3. Feeling pain in the wrists or at the back of the neck while using a smartphone.

- Strongly disagree
- Disagree
- Weakly disagree
- Weakly agree
- Agree
- Strongly agree

4. Won’t be able to stand not having a smartphone.

- Strongly disagree
- Disagree
- Weakly disagree
- Weakly agree
- Agree
- Strongly agree

5. Feeling impatient and fretful when I am not holding my smartphone.

- Strongly disagree
- Disagree
- Weakly disagree
- Weakly agree
- Agree
- Strongly agree

6. Having my smartphone in my mind even when I am not using it.

- Strongly disagree
- Disagree
- Weakly disagree
- Weakly agree
- Agree
- Strongly agree

7. I will never give up using my smartphone even when my daily life is already greatly affected by it.

- Strongly disagree
- Disagree
- Weakly disagree
- Weakly agree
- Agree
- Strongly agree

8. Constantly checking my smartphone so as not to miss conversations between other people on Twitter and Facebook.

- Strongly disagree
- Disagree
- Weakly disagree
- Weakly agree
- Agree
- Strongly agree

9. Using my smartphone longer than I had intended.

- Strongly disagree
- Disagree
- Weakly disagree
- Weakly agree
- Agree
- Strongly agree

10. The people around me tell me that I use my smartphone too much.

- Strongly disagree
- Disagree
- Weakly disagree
- Weakly agree
- Agree
- Strongly agree

***Section 3 : Attention deficit hyperactivity disorder self-report scale (ASRS-v1.1)***

1. How often do you have trouble wrapping up the final details of a project, once the challenging parts have been done?

- Never
- Rarely
- Sometimes
- Often
- Very often

1. How often do you have difficulty getting things in order when you have to do a task that requires organization?

- Never
- Rarely
- Sometimes
- Often
- Very often

1. How often do you have problems remembering appointments or obligations?

- Never
- Rarely
- Sometimes
- Often
- Very often

1. When you have a task that requires a lot of thought, how often do you avoid or delay getting started?

- Never
- Rarely
- Sometimes
- Often
- Very often

1. Put things off until the last minute. How often do you fidget or squirm with your hands or feet when you have to sit down for a long time?

- Never
- Rarely
- Sometimes
- Often
- Very often

1. How often do you feel overly active and compelled to do things, like you were driven by a motor?

- Never
- Rarely
- Sometimes
- Often
- Very often

1. How often do you make careless mistakes when you have to work on a boring or difficult project?

- Never
- Rarely
- Sometimes
- Often
- Very often

1. How often do you have difficulty keeping your attention when you are doing boring or repetitive work?

- Never
- Rarely
- Sometimes
- Often
- Very often

1. How often do you have difficulty concentrating on what people say to you, even when they are speaking to you directly?

- Never
- Rarely
- Sometimes
- Often
- Very often

1. How often do you misplace or have difficulty finding things at home or at work?

- Never
- Rarely
- Sometimes
- Often
- Very often

1. How often are you distracted by activity or noise around you?

- Never
- Rarely
- Sometimes
- Often
- Very often

1. How often do you leave your seat in meetings or other situations in which you are expected to remain seated?

- Never
- Rarely
- Sometimes
- Often
- Very often

1. How often do you feel restless or fidgety?

- Never
- Rarely
- Sometimes
- Often
- Very often

1. How often do you have difficulty unwinding and relaxing when you have time to yourself?

- Never
- Rarely
- Sometimes
- Often
- Very often

1. How often do you find yourself talking too much when you are in social situations?

- Never
- Rarely
- Sometimes
- Often
- Very often

1. When you’re in a conversation, how often do you find yourself finishing the sentences of the people you are talking to, before they can finish them themselves?

- Never
- Rarely
- Sometimes
- Often
- Very often

1. How often do you have difficulty waiting your turn in situations when turn taking is required?

- Never
- Rarely
- Sometimes
- Often
- Very often

1. How often do you interrupt others when they are busy?

- Never
- Rarely
- Sometimes
- Often
- Very often

Any comments

………………………………………………………………………………………………

Thank you for completing this surve

*Mandatory Questions

Tip: The questionnaire includes skip Logic questions
